# Oxyphosphate Cements

**Published:** 1901-03-15

**Authors:** Herman Fleck


### OXYPHOSPHATE CEMENTS.

So little is known regarding the composition and chemical structure of oxyphosphate plastics, that no definite statements beyond those which are justified by well-known laws of chemistry, can at present be made with safety. But if the very rudiments of this science are to be attacked and doubted, then further investigation leads to conclusions born of mental gymnastics which the careful thinker refuses to follow.

In the *Pacific Dental Gazette*, Vol. VIII, p. 370, Dr. J. Foster Flagg has created a "New Departure Creed," which to any one acquainted with the beaten paths of scientific thought can not fail to call forth surprise. The section referred to, which relates to the chemistry of oxyphosphates, runs thus:

"It is not to be denied that the nomenclature of dentistry is to be credited with descriptive, concise practicality, rather than with 'scientific accuracy,' but there is no warrant for the almost universal name 'Oxyphosphate' as given to those cements which are the nitrated oxid of zinc products, such as Poulson's, Fletcher's Justi's, Harvard, Dawson's, Hammond's and others of that class."

With reference to this statement let us ask, what is the meaning of "oxy?" To the chemist it has a twofold meaning. For his own convenience the latter has arranged the various elements in groups, which are termed "radicals." A radical may consist of one element O, divalent oxygen, or of two or more elements HO, monovalent hydroxly composed of hydrogen and oxygen. The relation of the latter to the former is readily seen in the compound H-O-H, water. Where either of these groups is present in the molecule the term

"oxy" may properly be applied. The chief factor in formation of either is water or some closely allied compound. Salts of zinc and aluminium are both capable of reacting with water to form hydrates or oxy salts, and that this is especially true of the compounds used in dentistry is proven by the fact that the liquids used contain from 10 to 30 percent water, and that when zinc oxid and phosphoric acid combine water is liberated in the reaction. What becomes of the water from these two sources? Experiments conducted by me show that the greater portion of it is recombined and bound fast. There is only one explanation. The water has combined to form an oxy or hydroxy phosphate. In this instance then, it will be acknowledged that "descriptive, concise practicality" and "scientific accuracy" are identical.

A looser sense of the term oxyphosphate would cover a phosphate of zinc containing a quantity of uncombined oxid of zinc acting as concrete or in very loosely bound form.

This is the case with a mixture made of nearly every powder and liquid at present in the market. A plastic mixture made from the above products contains in the neighborhood of three times more zinc oxid than the quantity required by chemical laws to neutralize the available phosphoric acid. In every sense then, the term oxyphosphate is applicable, unless Dr. Flagg be right in his newly-made discovery concerning the composition of zinc oxid made from the nitrated product. Let us consider the keynote of page 370 and this paper.

"There is no oxid of zinc in the powders of any properly prepared zinc phosphate"—not that it might not be possible to again make oxid of zinc from nitrated oxid, but that no such *transformation is made* by the processes used in making "zinc phosphate" powder.

There is considerable ambiguity and lack of scientific accuracy in this. Considering zinc phosphate as the compound containing three atoms of zinc, two of phosphorus and eight of oxygen, I fully agree with Dr. Flagg. Furthermore, there are a number of possible cases in which no oxid of zinc could possibly be present in an improperly prepared zinc phosphate. That this is not meant follows from the remainder of the sentence. We are left to suppose that the term "zinc phosphate" applies either to a mixture made from the liquid with a powder no longer zinc oxid, or that the term is applied to the powder which at first was oxid of zinc, but by treatment with nitric acid and subsequent ignition changed to a compound, the nature of which Dr. Flagg leaves us entirely in doubt.

As one is the logical result of the other it matters little which is given preference. The fact remains that Dr. Flagg has put himself in direct opposition to generally accepted views.

"We are told by chemists that this result is again 'oxid of zinc,' but this powder has nothing in common with that from which it was made; its 'feel' between the fingers is entirely different; its color is light yellow instead of white; its weight, in equal bulk is more than twice that of oxid of zinc, while its product with phosphoric acid is utterly distinct from that of the oxid."

There are different forms of many chemical compounds, both inorganic and organic.

Groups of oxid having the same composition chemically show different crystalline structure, different solubilities and gravities. The same may be said of the very elements which compose these compounds. Carbon exists in the forms of diamonds and graphite—

total opposites; one brilliant, hard and transparent, the other black or gray, soft enough to use as a lubricant and opaque. Phosphorus exists in several allotropic forms, and so does silicon. Titanium and aluminium oxides exist in different modifications, etc., etc.

That zinc oxid from the nitrate shows physical properties somewhat distinct from that obtained from other sources is surprising only to one unacquainted with the above facts. Furthermore, if zinc oxid prepared from the nitrate is not zinc oxid it must be a modification of the nitrate and would not contain the calculated quantity of zinc which the oxid requires.

Nitrate of zinc requires 34.39 percent zinc; oxid of zinc requires 80.24 percent zinc. The compound suggested by the idea of Dr. Flagg must have a percentage of zinc lying between these two extremes. Such is not the case. I have personally analyzed the powders accompanying the Poulson, Justi and Harvard cements, and with the exception of Harvard, which is a compounded powder of rather complex nature, the results show them to be fairly pure zinc oxid. But whether they had their origin in the nitrate I have no way of telling, not being in a position to ask the manufacturers to reveal their business secrets.

It is very unsafe to venture an opinion concerning chemical composition on the meagre data cited by Dr. Flagg.—*Herman Fleck, in Dental Brief.*

---

Dr. Walton, in *Items of Interest*, recommends 25 percent Pyrozone, applied on cotton, to assist in removing nerve broaches broken off in pulp canals. The application should remain for several days. [It is likely to rust the broach and make it brittle, and may discolor the tooth.—Ed.

